# Perceptions and Patterns of Cigarette and E-Cigarette Use among Hispanics: A Heterogeneity Analysis of the 2017–2019 Health Information National Trends Survey

**DOI:** 10.3390/ijerph18126378

**Published:** 2021-06-12

**Authors:** Stephanie Cardona, Rose Calixte, Argelis Rivera, Jessica Yasmine Islam, Denise Christina Vidot, Marlene Camacho-Rivera

**Affiliations:** 1 Department of Community Health Sciences, SUNY Downstate Health Sciences University, Brooklyn, NY 11203, USA; stephanie.cardona@downstate.edu; 2 Department of Epidemiology and Biostatistics, SUNY Downstate Health Sciences University, Brooklyn, NY 11203, USA; rose.calixte@downstate.edu; 3 Department of Medicine, Mount Sinai Icahn School of Medicine, New York, NY 10009, USA; argelis.rivera@mountsinai.org; 4 Cancer Epidemiology Program, H. Lee Moffitt Cancer Center and Research Institute, Tampa, FL 33612, USA; jessica.islam@moffitt.org; 5 Sylvester Comprehensive Cancer Center, School of Nursing and Health Studies, University of Miami, Miami, FL 33146, USA; dvidot@med.miami.edu

**Keywords:** smoking, cigarettes, e-cigarettes, Hispanic, health information

## Abstract

There are documented disparities in smoking behaviors among Hispanic adults in the U.S., but little is known about patterns of e-cigarette use. Using data from the HINTS 5 cycle 1–3, we examined cigarette and e-cigarette history and current use, as well as perceptions of the dangers of e-cigarette use relative to cigarette use. Primary predictors were Hispanic ethnic group, gender, age, education, income, and English language proficiency. Binary outcomes were modeled using the logit link, and multinomial outcome variables were modeled using generalized logit model. Fifty-three percent of participants were Mexican, 8% Puerto Rican, 4% were Cuban, and 35% identified as other Hispanics. Of the 1618 respondents, 23% were former cigarette smokers and 10% were current cigarette smokers. Twenty percent reported history of electronic cigarettes and 4% reported current use. In multivariable models, Hispanic women were significantly less likely to report ever being smokers compared to Hispanic men (aOR = 0.61, 95% CI = 0.42, 0.88). Puerto Ricans were 2.4 times as likely to report being current smokers (95% CI = 1.11, 5.11) compared to Mexicans. Among Hispanics, significant differences in e-cigarette and cigarette use behaviors emerged by gender, age, ethnicity, and cancer history, with implications for tailoring smoking prevention and cessation messages.

## 1. Introduction

Cancer is the leading cause of mortality among Hispanic and Latino populations within the Unites States [1]. Overall, lung cancer is the second most common cancer in both men and women, and represents approximately 25% of cancer-related deaths [2]. However, lung cancer is also the leading cause of death for Hispanic men and the second most common cause of death for Hispanic women [1,3]. Despite a lower incidence and mortality from lung cancer as a result of earlier intervention, detection, and smoking cessation over recent years, Hispanics and Latinos comprise a large number of heterogenous subpopulations that vary from the general population due, in part, to social, demographic, and environmental factors [4]. Although Hispanics have a 25% reduced incidence and 35% reduced mortality from cancer compared to non-Hispanic whites, combined data of Hispanics/Latinos as a whole can fail to acknowledge the essential heterogeneity within various subpopulations—including variable cancer risk [5]. In one study examining cancer-related deaths in disaggregated Hispanic subgroups that occurred from 2004–2014, mortality rates were higher in Cuban and Puerto Rican compared to all Hispanics combined, whereas Mexicans had very similar rates [5,6]. Among patients diagnosed with potentially curable stage 1 non-small cell lung cancer, Hispanics have worse all-cause mortality, lung-cancer-specific survival, and are less likely to undergo surgical resection compared to whites [3]. Notably, Hispanic patients who chose to undergo surgical resection had a similar survival rate compared to whites, suggesting that there are nonbiological factors engendering cancer disparities among Hispanics. Whether these ethnic disparities are patient-related or patient–physician-related, it is evident there is a need to further investigate the factors influencing cancer-related decisions among Hispanics.

The process by which the public searches for and obtains health-related information influences health literacy and decision-making related to healthcare. Nowadays, an increasingly common method for obtaining health-related information across different racial and ethnic groups is the internet [7,8]. Even the current COVID-19 pandemic has highlighted a higher interest in mobile health monitoring among patients with chronic health conditions when compared to healthier individuals [9]. Similarly, another study demonstrated a large prevalence of health-related internet use among cancer survivors, suggesting that more medically vulnerable groups frequently turn to the internet to seek health information [10]. According to a recent study conducted by our group that analyzed data collected from the Health Information National Trends Survey (HINTS), 80% of U.S. adult respondents have utilized the internet to look up health-related information, and 43% have used the internet to search for a doctor [11]. Disparities among health-information-seeking internet use and participation in online health interventions becomes more discernable while observing different educational levels, age groups, socioeconomic statuses, and languages [8,11,12]. Furthermore, closer analysis of different Hispanic subgroups seeking cancer-related health information online showed that, among these subgroups, there is also variability associated with trust in cancer information across various platforms [13]. Cubans and Puerto Ricans were two times more likely to trust printed media health information sources than Mexican Americans were. On the other hand, older Hispanics were found to be three times more likely to trust cancer-related information from religious sources compared to young adult Hispanics. Differences in perceptions of online health information between never, former, and current tobacco smokers show that current smokers are less likely to be trusting of health information sources, less likely to use the internet, and less trusting of medical professionals [13,14]. Current smokers are also less likely to have accurate beliefs related to smoking risks when compared to non- and former smokers [15].

Approximately 13% of Hispanic men and 7% of Hispanic women are current cigarette smokers [16]. Cigarette smoking is causally associated with multiorgan diseases, including diabetes mellitus, chronic obstructive pulmonary disease, cardiovascular disease, rheumatoid arthritis, lung cancer, and colorectal cancer [17]. Despite a decline in smoking incidence and mortality, disparities in tobacco use continue to exist among different racial and ethnic groups [17]. Over recent years, electronic cigarettes (e-cigarettes) have gained popularity as an alternative to cigarette smoking. Although a lot remains to be understood, e-cigarettes are commonly perceived as less harmful compared to cigarette smoking by the general public [18]. Prior studies have sought out to understand smoking-related patterns of belief between nonsmokers and current smokers, or understanding smoking behaviors in distinct geographic areas [13,14,18,19,20]. However, there has been no singular study examining smoking-related patterns of belief among Hispanic subgroups across the U.S. Understanding smoking beliefs and patterns of use within different stratified Hispanic populations could elucidate specific themes for future intervention and efforts to promote smoking literacy and prevention.

The objective of the present study is to identify social and demographic patterns of heterogeneity in cigarette and e-cigarette use among different Hispanic subgroups and recognize some of the perceptions held with regards to their consumption. In order to accomplish this, weighted survey data from the nationally representative Health Information National Trends Survey (HINTS) across three cycles (HINTS 5 Cycles 1–3) was incorporated to study trends among self-identifying Hispanic and Latino participants in the United States.

## 2. Materials and Methods

HINTS is a publicly available survey of knowledge, attitudes toward, and use of cancer- and health-related information, providing a representative sample of the noninstitutionalized adult population of the United States [21]. The sampling frame for HINTS 5 consisted of a database of addresses used by Marketing Systems Group (MSG) to provide random samples of address, where all nonvacant resident addresses in the US are present on the MSG database, including post office boxes and seasonal addresses [22]. HINTS includes quality assurance questions to monitor for the duplication of households in the sampling frame. The analytic sample was restricted to participants who self-identified as Hispanic or Latino (either U.S. born or foreign-born) from the following three HINTS cycles: HINTS 5 cycle 1, collected from January 2017 to May 2017; HINTS 5 cycle 2, collected from January 2018 to May 2018; and HINTS 5 cycle 3 was collected from January 2019 to May 2019, to yield a total sample of 1618. The sampling design for the HINTS survey has been described extensively [21]. The overall response rates were 32.4% for HINTS 5 cycle 1, 32.985% for HINTS 5 cycle 2, and 30.3% for HINTS 5 cycle 3, based on self-administered questionnaires. HINTS 5 cycle 3 was also supplemented by a web pilot survey. Overall, we have 427 participants from cycle 1, 460 participants from cycle 2, and 730 participants from cycle 3. The cycle 3 participants were further distributed into three domains: 457 participants from the paper survey, 113 participants from the web pilot, and 160 participants from the web bonus survey.

The primary outcomes of interest are: cigarette use (yes vs. no), measured by “have you ever smoke 100 cigarettes or more in your lifetime?”; smoking status (current, former, never), measured by combining “have you ever smoked 100 cigarettes or more in your lifetime?” and “do you smoke now?”; current smoking status (dichotomized smoking status); electronic cigarette (e-cigarette) use (yes/no), measured by “have you ever used an e-cigarette even one or two times?” and “compared to smoking cigarette, would you say e-cigarettes are?” Response choices were: “much less harmful”, “less harmful”, “just as harmful”, “more harmful”, “much more harmful”, “I have never heard about e-cigarette”, and “I don’t know enough about these products”. These cigarette and e-cigarette use variables have been previously utilized in other national surveys, such as the National Health Interview Survey [23]. We reduced the number of categories to 4 by collapsing “much less harmful” with “less harmful”, “more harmful” with “much more harmful”, and “I have never heard about e-cigarette” with “I don’t know enough about these products”. The predictors of interest are gender (male vs. female), age (18–34, 35–49, 50–64, ≥ 65), ethnicity (Cuban, Mexican, Puerto Rican, Other Hispanics), education (less than high school, high school graduate, some college or more), income (<USD 35,000, USD 35,000–<USD 75,000, ≥USD 75,000), English language proficiency (well and very well vs. not well and not at all), personal history of cancer (yes vs. no), and family history of cancer (yes vs. no). The covariate of interest is HINTS cycle. Predictors and covariates were selected a priori based on review of existing literature on demographic predictors of cigarette and e-cigarette use [24,25,26].

We fit a multivariable logistic regression to model the outcome variables. All the analyses were adjusted by the HINTS cycle to measure changes in outcome variables per cycle. Binary outcomes were modeled using the logit link, and multinomial outcome variables were modeled using generalized logit model. For smoking status, we used those who have never smoked as the reference category for the multinomial model. For how harmful e-cigarettes are compared to cigarettes, we used “don’t know” as the reference category to fit the multinomial model.

Missing responses were imputed using the hot-deck imputation method to preserve the distribution of observed responses. Each observation with missing data was imputed 20 times, and the imputed weight was recalibrated to preserve the national representative survey weight. Hot-deck imputation is the preferred imputation method for HINTS; additional details may be found in the methodology report [22,27]. All analyses were done using SAS 9.4^®^ (SAS Institute Inc., Cary, NC, USA) using complex survey methodology with jackknife replicate weights for accurate standard errors, with all analyses weighted to provide nationally representative estimates. Statistical significance level was set at α = 0.05.

## 3. Results

We present the summary statistics of the study sample in Table 1. The weighted proportion of Hispanic participants who self-identified as Mexican was 53.3%. Puerto-Ricans represented 8% of the participants. Cubans accounted for just about 4% of the responders. The remaining 35% were identified as other Hispanic. Just over 58% of the participants reported having some college education. Overall, about 64% of the participants who self-identified as Hispanic were between 18 and 49 years old. Just over 10% were 65 years of age and older. About 31% of the participants have household income less than USD 35,000. The demographic characteristics of participants did not differ across HINTS cycles. Close to 90% of the responders reported a high level of English language proficiency. While 5% of the responders have been diagnosed with cancer, close to 55% of the participants have a family history of cancer. Close to 23% of the responders have been smokers. However, only 10% of the responders are current smokers. Just about 20% of the participants reported use of electronic cigarettes. However, only about 4% of the responders are current e-cigarette users. About 14% of responders reported that e-cigarettes are less harmful than cigarettes, 36.1% of responders think that e-cigarettes are just as harmful as cigarettes, and about 13% of the responders think that e-cigarettes are more harmful than cigarettes. The remaining participants (~33%) stated that they either have never heard of e-cigarettes or they did not know enough about e-cigarettes.

### 3.1. Multivariable Logistic Model Results for Binary Responses for Cigarette and E-Cigarette Use

We present the result of the multivariable binary logistic regression model for cigarette use, current cigarette use, and e-cigarette use in Table 2. The result of the multivariable binary logistic regression, predicting variables that are associated with ever smokers, indicates that Hispanic females had significantly reduced odds of being ever smokers (adjusted odds ratio (aOR) = 0.61, 95% CI = 0.42, 0.88) compared to Hispanic males. Hispanics 35 years and older were more likely to have been ever smokers compared to Hispanics who were 18–34 years old. More specifically, Hispanics 35–49 years old were 2.9 times (95% CI = 1.69, 4.99) more likely to have ever been smokers. Hispanics 50–64 years old were 3.15 times (95% CI = 1.86, 5.33) more likely to have ever been smokers. Hispanics 65 years and older were 5.13 times (95% CI = 2.90, 9.06) more likely to have ever been smokers. Hispanics who reported not speaking English well had significantly reduced odds of having ever been smokers (aOR = 0.42, 95% CI = 0.20, 0.85) compared to those who speak English well. High school graduates of Hispanic origin were less likely to have ever been smokers (aOR = 0.57, 95% CI = 0.34, 0.95) compared to participants who had some college education. No other variables were significant predictors of ever smoker. Most importantly, the odds ratio for ever smokers has not changed over the HINTS cycles.

When smoking status was dichotomized as current smokers (yes vs. no), the set of predictors slightly changed. Hispanics 35–49 years old were 2.24 times (95% CI = 1.05, 4.74) more likely to be current smokers compared to Hispanics 18–34 years old. Puerto Ricans were 2.39 times (95% CI = 1.11, 5.11) more likely to be current smokers vs. Mexicans. Lastly, Hispanics who reported not speaking English well had significantly reduced odds of being current smokers (aOR = 0.18, 95% CI = 0.08, 0.50). No other variables significantly predict current smokers among Hispanic HINTS participants. Most importantly, the odds ratio for current smokers has not changed over the HINTS cycles.

The result of the multivariable logistic regression model for e-cigarette use indicates that Hispanic females were significantly less likely to have ever used e-cigarettes (aOR = 0.54, 95% CI = 0.30, 0.96). Hispanics 35 years and older were significantly less likely to have ever used e-cigarettes compared to Hispanics 18–34 years old (aOR = 0.28, 95% CI = 0.11, 0.69, aOR = 0.09, 95% CI = 0.04, 0.21, and aOR = 0.07, 95% CI = 0.02, 0.24, age 35–49, 50–64, and ≥65, respectively). Hispanics who reported not speaking English well had significantly reduced odds of being current smokers (aOR = 0.12, 95% CI = 0.03, 0.51). Participants with a history of cancer have significantly greater odds of e-cigarette use (aOR = 3.45, 95% CI = 1.04, 11.50). Compared to cycle 1, participants in cycle 2 were 2.50 times (95%CI = 1.15, 5.46) and participants of cycle 3 were 2.80 times (95% CI = 1.18, 6.670) more likely to have used e-cigarettes, indicating increasing exposure to e-cigarettes over the years. No other variables of interest were associated with e-cigarettes use.

### 3.2. Multivariable Multinomial Logistic Model Results for Smoking Status

We present the results of the multinomial regression model for smoking status in Table 3. Overall, in the multinomial model, gender is not associated with smoking status. Compared to Hispanics 18–34 years old, Hispanics between age 35 and 64 have increased odds of being current smokers vs. never smokers (aOR = 2.52, 95% CI = 1.19, 5.33 and aOR = 2.15, 95% CI = 1.04, 4.44 for age 35–49 and 50–64, respectively). Compared to Hispanics 18–34 years old, Hispanics ≥ 35 years old have increased odds of being former smokers vs. never smokers (aOR = 3.44, 95% CI = 1.60, 7.39, aOR = 4.38, 95% CI = 2.22, 8.66, and aOR = 10.25, 95% CI = 5.14, 20.41 for age 35–49, 50–64, and ≥ 65, respectively). Compared to Mexicans, Puerto Ricans had significantly increased odds of being current smokers vs. never smokers (aOR = 2.34, 95% CI = 1.09, 5.02). Compared to Hispanics with some college education, high school graduates had significantly reduced odds of being former smokers vs. never smokers (aOR = 0.43, 95% CI = 0.25, 0.76). Hispanics reporting not speaking English well had significantly reduced odds of being current smokers vs. never smokers (aOR = 0.17, 95% CI = 0.06, 0.47). Hispanics with a family history of cancer had significantly increased odds of being former smokers vs. never smokers (aOR = 1.62, 95% CI = 1.04, 2.52).

### 3.3. Multivariable Multinomial Logistic Regression Model Comparing Perceptions around Harm of E-Cigarettes Compared to Cigarettes

We present the result of the multinomial regression model for how harmful an e-cigarette is compared to cigarettes in Table 4. Women were significantly less likely to say an e-cigarette is less harmful than a cigarette, as opposed to “I don’t know enough about e-cigarettes” (aOR = 0.34, 95% CI = 0.19, 0.63). Hispanics 35–64 years old were significantly less likely to say an e-cigarette is less harmful than a cigarette, as opposed to “I don’t know enough about e-cigarettes” (aOR = 0.12, 95% CI = 0.05, 0.27, aOR = 0.12, 95% CI = 0.03, 0.31 for age 35–49, and 50–64, respectively). Hispanics who did not speak English well were significantly less likely to say that an e-cigarette is less harmful than a cigarette, as opposed to “I don’t know enough about e-cigarettes” (aOR = 0.22, 95% CI = 0.07, 0.68). Hispanics with a history of cancer were significantly more likely to say that an e-cigarette is less harmful than a cigarette, as opposed to “I don’t know enough about e-cigarettes” (aOR = 5.00, 95% CI = 1.48, 16.90). Participants in cycle 2 and participants in cycle 3 were less likely to say that an e-cigarette is less harmful than a cigarette, as opposed to “I don’t know enough about e-cigarettes” (aOR = 0.11, 95% CI = 0.06, 0.21 and aOR = 0.18, 95% CI = 0.08, 0.42 for cycle 2 and cycle 3, respectively). Comparing those who rated e-cigarettes just as harmful as cigarettes vs. those who said they do not know, no participant characteristics were associated with the response level. However, participants in cycle 2 and participants in cycle 3 were less likely to say that an e-cigarette is less harmful than a cigarette, as opposed to “I don’t know enough about e-cigarettes” (aOR = 0.25, 95% CI = 0.15, 0.43 and aOR = 0.24, 95% CI = 0.15, 0.40 for cycle 2 and cycle 3, respectively). Participants 35–49 years old had significantly reduced odds of stating an e-cigarette is more harmful than a cigarette vs. “don’t know” compared to participants 18–34 years old (aOR = 0.41, 95% CI = 0.18, 0.94). Additionally, participants in cycle 2 had significantly reduced odds of stating an e-cigarette is more harmful than a cigarette vs. “don’t know” compared to participants in cycle 1 (aOR = 0.38, 95% CI = 0.19, 0.77). No other variables significantly predict the odds of choosing e-cigarettes as more harmful than cigarettes vs. “I don’t know enough about e-cigarettes”.

## 4. Discussion

To our knowledge, this is the first study that has examined Hispanic subgroups with a history of cigarette and e-cigarette use and their perceptions of smoking with a nationally representative sample of Latinos. In this study, we demonstrated how, among various Hispanic subpopulations, there were significant differences observed regarding current smoker status. We observed that that Puerto Ricans are largely more likely to be current smokers when compared to Mexicans or Mexican-Americans. This is consistent with previous observations that Puerto Rican males have a high cigarette smoking prevalence when compared to U.S. non-Hispanic white males, while Mexican-American males have a similar smoking prevalence to non-Hispanic white males [28]. When taking cancer disparities into consideration, this finding supports the idea that certain groups may be at a higher risk of susceptibility when considering education, smoking awareness, and language [11,12].

We found that Hispanics who reported not speaking English well had significantly reduced odds of having ever been smokers compared to those who spoke English well. Furthermore, high school graduates of Hispanic origin were less likely to have ever been smokers [29]. These findings are consistent with previous studies demonstrating the influence of acculturation into the United States and the English language on Hispanic health status [28,30,31,32]. Individuals that are more acclimated and accepting of U.S. culture are understood to be more likely to be current smokers, along with practicing other unhealthy behaviors, including a greater likelihood of alcohol intake and a higher BMI [32,33]. Our findings also indicate that the influence of acculturation extends into education. Accordingly, we found that Hispanics with a high school graduate education level had reduced odds of being current smokers when compared to Hispanics with some college level education. This is an interesting finding when considering that, overall, higher education levels have been previously associated with decreased smoking behavior [15,28,34]. As our analyses utilize more recent data than previously published studies, these findings may reflect a shift in the demographic composition of Hispanic smokers.

Another important finding in this study was the change in smoking status among Hispanics overall in recent years [35]. While close to 23% of the responders have been smokers, only 10% are current smokers. A similar pattern was observed with the use of e-cigarettes. While about 20% of the responders reported ever using electronic cigarettes, only about 4% are current e-cigarette users. This might implicate that, with time and increased health information seeking, Hispanics have become increasingly aware of the dangers and potential side effects that can result from both cigarette and e-cigarette smoking. Additional studies using nationally representative samples have documented similar patterns of decreased e-cigarette consumption within the U.S. [36]. Further, recent implementation of e-cigarette smoke-free policies across states, excise taxes on e-cigarettes, and raising the tobacco legal purchasing age to 21 years may have impacted changes in e-cigarette use across HINTS cycles [37]

Our study also found that women had significantly reduced odds of being ever smokers and e-cigarette smokers. This is consistent with previous research demonstrating that females, overall, tend to have more accurate smoking-related risk beliefs [14]. While less is known about gender differences in e-cigarette smoking within Latinos, the patterns are consistent with other studies of smoking within Latino epidemiological cohort studies [28].

Our study findings should be interpreted in the context of the study’s strengths and limitations. Strengths of our study include the use of a nationally representative U.S. sample to examine smoking-related patterns and beliefs among Hispanics. At large, the use of HINTS data permitted expansion of previous research on more restricted geographical areas within the United States. The application of this large cohort for our study improves generalizability and further allowed us to examine more closely the patterns and beliefs among specific Latino subgroups. When evaluating for smoking-related perceptions, we found it important to not only assess cigarette smoking behaviors, but also electronic cigarette behaviors as well, considering their relevance in today’s world.

While our present study has illuminated and confirmed existing disparities within Hispanic ethnic subgroups across the U.S., this study has limitations that should be considered when interpreting its reported findings. For one, in our study, we were only able to investigate some, not all, Hispanic ethnic subgroups. However, Hispanics of Mexican, Puerto Rican, and Cuban origin represent the three largest Hispanic ethnicity groups in the US. Second, because the HINTS dataset is the result of cross-sectional self-reported data, recall bias, misclassification bias, and social desirability in response to certain questions cannot be excluded and must be taken into consideration as possible influences among respondents. Lastly, although the data were from a probability-based national sample utilizing a full sample weight and 50 replicate weights that calculated household-level and person-level weights, adjusted for household non-response, and calibrated the person-level weights to population control totals from the U.S. Census Bureau’s American Community Survey to correct for nonresponse and noncoverage biases, it is possible that estimates derived from Hispanic respondents in HINTS may not be representative of Hispanics in the U.S. overall.

## 5. Conclusions

In this study, we observed several significant smoking-related disparities among different Hispanic and Latino ethnic subgroups across the Unites States. We expanded on previous studies by noting the high smoking prevalence among Puerto Ricans when compared to Mexicans. Puerto Rican adults should be considered a priority group when targeting smoking prevention and smoking cessation efforts, and should receive increased attention for cancer screening, given the smoking rates we observed [38]. When considering the influence of acclimation into U.S. culture on worse health outcomes, our study found that Hispanics who reported not being fluent in the English language had significantly reduced odds of being smokers. With regards to the relationship between education and smoking status behaviors, our findings suggest acculturation continues to influence smoking patterns among Hispanics [39]. Between Hispanic males and females, we found that there were no statistically significant differences in current smoking status, although women had significantly reduced odds of being ever smokers. These results indicate that smoking-related beliefs and behaviors continue to have a major role in the Hispanic community that need to be further examined according to the risks correlated by different ethnic subgroups. Given the increasing evidence linking e-cigarettes to cancer outcomes, as well e-cigarette or vaping product use-associated lung injury (EVALI), significant attention should be paid to reducing e-cigarette initiation among Hispanics, in particular, young adults [40,41,42]. While the prevalence of smoking at large continues to decline, it is an important public health responsibility for future studies, medical professionals, and health interventions to acknowledge these differences among Hispanic subpopulations. By targeting specific groups with relevant methods for providing accessible and trustworthy health-related smoking information, we may be able to reduce and intervene on these health inequities that, in the long run, influence the presence of health comorbidities and cancer risk.

## Figures and Tables

**Table 1:** Demographic and smoking characteristics of Hispanic respondents in 2017–2019 HINTS (*N* = 1618).

Characteristics	Frequency (%)	Weighted Percent (SE)
*N* total	1618	
**Age**		
18–34	288 (17.8%)	30.4 (2.66)
35–49	430 (26.6%)	33.7 (2.44)
50–64	477 (29.5%)	24.0 (1.75)
65–74	258 (16.0%)	6.5 (0.65)
≥75	138 (8.5%)	4.0 (0.51)
Missing	27 (1.7%)	1.4 (0.55)
**Gender**		
Male	659 (40.7%)	44.7 (2.18)
Female	874 (54.0%)	48.5 (2.07)
Missing	85 (5.3%)	6.8 (2.55)
**Ethnicity**		
Cuban	75 (4.6%)	4.1 (1.23)
Mexican	773 (48.8%)	53.3 (2.34)
Puerto Rican	166 (10.3%)	8.0 (1.16)
Other Hispanics	604 (37.3%)	34.6 (2.20)
**Education Level**		
Less than High School	266 (16.4%)	16.6 (1.68)
High School Graduate	319 (19.7%)	24.4 (2.47)
Some College	492 (30.4%)	38.4 (2.30)
College Graduate or More	521 (32.2%)	20.0 (1.19)
Missing	20 (1.2%)	0.6 (0.24)
**Marital Status**		
Married/Living Together	870 (53.8%)	52.4 (2.16)
Divorced/Separated	277 (17.2%)	7.5 (0.74)
Widowed	137 (8.5%)	2.7 (0.37)
Single	304 (18.8%)	36.1 (2.23)
Missing	30 (1.9%)	1.3 (0.53)
**Income Level**		
<USD 20,000	370 (22.9%)	18.5 (1.70)
USD 20,000–< USD 35,000	233 (14.4%)	12.7 (1.47)
USD 35,000–< USD 50,000	215 (13.3%)	16.6 (1.98)
USD 50,000–< USD 75,000	250 (15.5%)	16.4 (1.91)
≥USD 75,000	433 (26.8%)	27.3 (2.27)
Missing	117 (7.2%)	8.5 (2.63)
**English Speaking Proficiency**		
Very Well	1084 (67.0%)	69.1 (2.02)
Well	304 (18.8%)	17.9 (1.77)
Not Well	170 (10.5%)	9.5 (1.16)
Not at All	36 (2.2%)	2.7 (0.78)
Missing	24 (1.5%)	0.8 (0.25)
**Personal History of Cancer**		
Yes	131 (8.1%)	5.4 (1.09)
No	1482 (91.6%)	94.5 (1.09)
Missing	5 (0.3%)	0.1 (0.09)
**Family History of Cancer**		
Yes	949 (58.7)%)	54.5 (3.04)
No	490 (30.3%)	31.8 (2.23)
Don’t Know	154 (9.5%)	12.5 (2.67)
Missing	25 (1.6%)	1.2 (0.42)
**HINTS 5**		
Cycle 1	427 (26.4%)	19.1 (0.28)
Cycle 2	461 (28.5%)	19.5 (0.31)
Cycle 3	730 (45.1%)	61.4 (0.36)
**HINTS 5 Cycle 3**		
Cycle 3 Paper	457 (28.2%)	20.6 (0.21)
Cycle 3 Web	113 (7.0%)	20.7 (0.25)
Cycle 3 Web Bonus	160 (9.9%)	20.1 (0.35)
**Smoke more than 100 Cigarette in Lifetime**		
Yes	456 (28.2%)	22.8 (1.87)
No	1155 (71.4%)	77.0 (1.88)
Missing	7 (0.4%)	0.2 (0.10)
**Smoking Status**		
Current	179 (11.1%)	10.0 (1.41)
Former	271 (16.8%)	12.5 (1.37)
Never	1155 (71.4%)	77.0 (1.88)
Missing	13 (0.8%)	0.5 (0.20)
**Ever Used e-Cigarette**		
Yes	205 (12.7%)	20.2 (2.51)
No	1396 (86.3%)	79.3 (2.51)
Missing	17 (1.1%)	0.5 (0.17)
**e-Cigarette Use Status**		
Current	40 (2.5%)	3.8 (1.20)
Former	164 (10.1%)	16.3 (2.29)
Never	1396 (86.3%)	79.3 (2.51)
Missing	18 (1.1%)	0.6 (0.17)
**Compared to smoking** **cigarette, would you say** **e-Cigarette are**		
Much less harmful	46 (2.8%)	4.8 (2.49)
Less harmful	139 (8.6%)	9.8 (1.43)
Just as harmful	556 (34.4%)	36.1 (2.35)
More harmful	113 (7.0%)	6.9 (1.26)
Much more harmful	112 (6.9%)	6.0 (1.21)
I’ve never heard of electronic cigarettes	85 (5.3%)	2.7 (0.43)
I don’t know enough about these products	498 (30.8%)	30.8 (2.17)
Missing	69 (4.3%)	2.9 (0.63)

**Table 2:** Multivariable logistic model results for binary responses for cigarette and e-cigarette use (N = 1618).

Variable	Have You Smoked More Than 100 Cigarette in Your Lifetime?		Do You Smoke Now?		Have You Ever Used an E-Cigarette, Even One or Two Times?	
	OR	95% CI	OR	95% CI	OR	95% CI
Gender (ref male)						
Female	**0.61**	(0.42, 0.88) **	0.60	(0.33, 1.09)	**0.54**	(0.30, 0.96) *
Age (years) (ref 18–34)						
35–49	**2.90**	(1.68, 4.99) ***	**2.24**	(1.05, 4.74) *	**0.28**	(0.11, 0.69) **
50–64	**3.15**	(1.86, 5.33) ***	1.82	(0.89, 2.85)	**0.09**	(0.04, 0.21) ***
≥65	**5.13**	(2.90, 9.06) ***	1.16	(0.47, 2.85)	**0.07**	(0.02, 0.24) ***
Hispanic Category	(ref Mexican)					
Puerto Rican	1.63	(0.82, 3.23)	**2.39**	(1.11, 5.11) *	**0.38**	(0.15, 0.97) *
Cuban	1.43	(0.49, 4.21)	1.51	(0.34, 6.77)	1.59	(0.24, 10.55)
Other Hispanics	1.58	(0.92, 2.72)	1.74	(0.77, 3.94)	0.87	(0.39, 1.95)
Education (ref some college or more)						
Less than High School	1.17	(0.57, 2.39)	2.43	(0.91, 6.52)	1.69	(0.59, 4.89)
High School Graduate	**0.57**	(0.34, 0.95) *	0.79	(0.39, 1.62)	1.26	(0.33, 4.74)
Income (ref ≥ USD 75,000)						
<USD 35,000	0.92	(0.98, 1.75)	1.04	(0.47, 2.27)	0.56	(0.26, 1.20)
USD 35,000–USD 74,999.99	0.66	(0.38, 1.16)	0.63	(0.26, 1.52)	0.70	(0.31, 1.57)
English Proficiency (ref well)						
Not Well	**0.42**	(0.20, 0.85) *	**0.18**	(0.08, 0.50) ***	**0.12**	(0.03, 0.51) **
Personal History of Cancer (ref no)						
Yes	1.66	(0.78, 3.51)	1.05	(0.42, 2.63)	**3.45**	(1.04, 11.50) *
Family History of Cancer (ref no)						
Yes	1.61	(0.98, 2.65)	1.54	(0.62, 3.86)	1.48	(0.72, 3.05)
HINTS 5 (ref cycle 1)						
Cycle 2	1.28	(0.82, 2.01)	1.82	(0.94, 3.54)	**2.50**	(1.15, 5.46) *
Cycle 3	0.97	(0.62, 1.54)	1.04	(0.50, 2.20)	**2.80**	(1.18, 6.70) *

* *p* ≤ 0.05, ** *p* ≤ 0.01, and *** *p* ≤ 0.001.

**Table 3:** Multivariable multinomial logistic model results for smoking status (*N* = 1618).

Variable	Current Cigarette User vs. Never User		Former Cigarette User vs. Never User	
	OR	95% CI	OR	95% CI
Gender (ref male)				
Female	0.56	(0.31, 1.00)	0.63	(0.38, 1.06)
Age (years) (ref 18–34)				
35–49	**2.52**	(1.19, 5.33) *	**3.44**	(1.60, 7.39) **
50–64	**2.15**	(1.04, 4.44) *	**4.38**	(2.22, 8.66) ***
≥65	1.70	(0.67, 4.31)	10.25	(5.14, 20.41) ***
Hispanic Category (ref Mexican)				
Puerto Rican	**2.34**	(1.09, 5.02) *	0.90	(0.33, 2.46)
Cuban	1.56	(0.33, 7.35)	1.27	(0.33, 4.93)
Other Hispanics	1.86	(0.80, 4.21)	1.33	(0.75, 2.36)
Education (ref some college of more)				
Less than High School	2.21	(0.81, 6.03)	0.59	(0.27, 1.28)
High School Graduate	0.70	(0.34, 1.47)	**0.43**	(0.25, 0.76) **
Income (ref ≥ USD 75,000)				
<USD 35,000	1.01	(0.45, 2.26)	0.89	(0.38, 2.09)
USD 35,000–USD 74,999.99	0.61	(0.25, 1.47)	0.78	(0.44, 1.39)
English Proficiency (ref well)				
Not Well	**0.17**	(0.06, 0.47) ***	0.72	(0.30, 1.74)
Personal History of Cancer (ref no)				
Yes	1.25	(0.49, 3.22)	1.90	(0.76, 4.79)
Family History of Cancer	(ref no)			
Yes	1.66	(0.67, 4.12)	**1.62**	(1.04, 2.52) *
HINTS 5 (ref cycle 1)				
Cycle 2	1.78	(0.92, 3.46)	0.88	(0.50, 1.53)
Cycle 3	1.03	(0.49, 2.18)	0.92	(0.54, 1.59)

* *p* ≤ 0.05, ** *p* ≤ 0.01, and *** *p* ≤ 0.001.

**Table 4:** Multivariable multinomial logistic regression model comparing perceptions around harm of e-cigarettes compared to cigarettes (*N* = 1618).

Variable	e-Cigarette Less Harmful vs. Don’t Know Enough about e-Cigarette		e-Cigarette Just as Harmful vs. Don’t Know Enough about e-Cigarette		e-Cigarette More Harmful vs. Don’t Know Enough about e-Cigarette	
	OR	95% CI	OR	95% CI	OR	95% CI
Gender (ref male)						
Female	**0.34**	(0.19, 0.63) ***	1.04	(0.66, 1.64)	1.10	(0.65, 1.86)
Age (years) (ref 18–34)						
35–49	**0.12**	(0.05, 0.27) ***	0.59	(0.28, 1.24)	**0.41**	(0.18, 0.94) *
50–64	**0.12**	(0.03, 0.31) ***	0.57	(0.22, 1.02)	0.57	(0.24, 1.36)
≥65	**0.03**	(0.01, 0.09) ***	0.50	(0.24, 1.04)	0.51	(0.21, 1.20)
Hispanic Category	(ref Mexican)					
Puerto Rican	0.68	(0.23, 2.08)	0.94	(0.36, 2.45)	1.23	(0.43, 3.53)
Cuban	0.56	(0.09, 3.31)	1.36	(0.25, 7.31)	1.43	(0.22, 9.52)
Other Hispanics	0.57	(0.26, 1.26)	0.79	(0.48, 1.32)	1.08	(0.52, 2.25)
Education (ref some college or more)						
Less than High School	0.72	(0.23, 2.22)	0.59	(0.31, 1.12)	0.44	(0.17, 1.10)
High School Graduate	1.40	(0.28, 7.01)	0.98	(0.61, 1.57)	0.93	(0.36, 2.40)
Income (ref ≥ USD 75,000)						
<USD 35,000	0.68	(0.21, 2.22)	0.51	(0.25, 1.04)	0.95	(0.41, 2.17)
USD 35,000–USD 74,999.99	0.65	(0.23, 1.80)	0.90	(0.44, 1.82)	1.03	(0.46, 1.82)
English Proficiency (ref well)						
Not Well	**0.22**	(0.07, 0.68) ***	0.71	(0.35, 1.45)	1.08	(0.42, 2.80)
Personal History of (ref no) Cancer						
Yes	**5.00**	(1.48, 16.90) ***	1.03	(0.42, 2.53)	1.24	(0.29, 5.30)
Family History of Cancer (ref no)						
Yes	1.30	(0.60, 2.82)	1.39	(0.82, 2.35)	1.10	(0.59, 2.02)
HINTS 5 (ref cycle 1)						
Cycle 2	**0.11**	(0.06, 0.21) ***	**0.25**	(0.15, 0.43) ***	**0.38**	(0.19, 0.77) **
Cycle 3	**0.18**	(0.08, 0.42) ***	**0.24**	(0.15, 0.40) ***	0.52	(0.25, 1.08)

* *p* ≤ 0.05, ** *p* ≤ 0.01, and *** *p* ≤ 0.001.

## Data Availability

Data for these analyses are publicly available through the National Cancer Institute’s Health Information National Trends Survey website, accessed here: https://hints.cancer.gov/data/download-data.aspx (access on 20 April 2021).

## Abbreviations

HINTS	Health Information National Trends Survey
U.S.	United States
BMI	body mass index
OR	odds ratio
aOR	adjusted odds ratio
95% CI	95% confidence intervals
SE	standard error
